# Genome‐wide DNA methylation profiling confirms a case of low‐level mosaic Kabuki syndrome 1

**DOI:** 10.1002/ajmg.a.62754

**Published:** 2022-04-06

**Authors:** Carolina Montano, Jacquelyn F. Britton, Jacqueline R. Harris, Jennifer Kerkhof, Benjamin T. Barnes, Jennifer A. Lee, Bekim Sadikovic, Nara Sobreira, Jill A. Fahrner

**Affiliations:** ^1^ National Human Genome Research Institute National Institutes of Health Bethesda Maryland USA; ^2^ Department of Genetic Medicine Johns Hopkins University School of Medicine Baltimore Maryland USA; ^3^ Department of Pediatrics Johns Hopkins University School of Medicine Baltimore Maryland USA; ^4^ Department of Neurogenetics Kennedy Krieger Institute Baltimore Maryland USA; ^5^ Molecular Genetics Laboratory, Molecular Diagnostics Division London Health Sciences Centre London Ontario Canada; ^6^ Greenwood Genetic Center Greenwood South Carolina USA; ^7^ Department of Pathology and Laboratory Medicine Western University London Ontario Canada

**Keywords:** DNA methylation, episignature, HLHS (hypoplastic left heart syndrome), Kabuki syndrome, *KMT2D*, mosaicism

## Abstract

Kabuki syndrome is a Mendelian disorder of the epigenetic machinery characterized by typical dysmorphic features, intellectual disability, and postnatal growth deficiency. Pathogenic variants in the genes encoding the chromatin modifiers KMT2D and KDM6A are responsible for Kabuki syndrome 1 (KS1) and Kabuki syndrome 2 (KS2), respectively. In addition, 11 cases of KS1 caused by mosaic variants in *KMT2D* have been reported in the literature. Some of these individuals display milder craniofacial and growth phenotypes, and most do not have congenital heart defects. We report the case of an infant with severe hypoplastic left heart syndrome with mitral atresia and aortic atresia (HLHS MA‐AA), pulmonary vein stenosis, and atypical facies with a somatic mosaic de novo nonsense variant in *KMT2D* (c.8200C>T, p.R2734*) identified on trio exome sequencing of peripheral blood and present in 11.2% of sequencing reads. KS was confirmed with EpiSign, a diagnostic genome‐wide DNA methylation platform used to identify epigenetic signatures. This case suggests that use of this newly available clinical test can guide the interpretation of low‐level mosaic variants identified through sequencing and suggests a new lower limit of mosaicism in whole blood required for a diagnosis of KS.

## 1 INTRODUCTION

Kabuki syndrome (KS) is a congenital syndrome characterized by typical dysmorphic features (long palpebral fissures with eversion of the lateral third of the lower eyelid, short columella with broad and depressed nasal tip, arched and broad eyebrows with sparseness or notching, prominent or cupped ears, and persistent fingertip pads), mild‐to‐moderate intellectual disability, and postnatal growth deficiency (Adam et al., 2019; Kuroki et al., 1981; Niikawa et al., 1981). Prevalence is estimated at 1 in 32,000 (Bögershausen et al., 2016; Niikawa et al., 1981). Congenital heart defects occur in 28%–80% of patients with KS, about half of which are left‐sided obstructive lesions (including aortic coarctation, aortic stenosis, bicuspid aortic valve, and hypoplastic left heart syndrome) (Digilio et al., 2017; Murakami et al., 2020). The KS phenotype changes over time: typical craniofacial features, and especially eye and eyebrow findings, are often not evident in the newborn period and become more easily recognizable between ages 3 and 12 years (Dentici et al., 2015; Vaux et al., 2005).

KS is caused by hypomorphic or inactivating variants in either one of two genes encoding enzymatic components of the epigenetic machinery with complementary writing and erasing functions. The two forms of KS exhibit highly similar phenotypes (Fahrner & Bjornsson, 2014). Kabuki syndrome 1 (KS1, MIM #147920) is an autosomal dominant disorder resulting from heterozygous typically de novo loss‐of‐function variants in *KMT2D*, which encodes an epigenetic histone H3K4 methyltransferase writer (Ng et al., 2010). Kabuki syndrome 2 (KS2 MIM #300867) is an X‐linked disorder caused by variants in the lysine demethylase *KDM6A*, an epigenetic eraser that removes trimethylation from H3K27 (Dentici et al., 2015; Lederer et al., 2012; Miyake et al., 2013). Pathogenic variants in *KMT2D* and *KDM6A* account for approximately 56%–75% and 5%–8% of KS cases, respectively (Bögershausen et al., 2016). Deficiency of *KMT2D* or *KDM6A* interferes with the opening of chromatin, affecting transcription of downstream targets across multiple cell types (Boniel et al., 2021; Fahrner & Bjornsson, 2019) and disrupting genomic DNA methylation (Aref‐Eshghi, Schenkel, Lin, Skinner, Ainsworth, Pare, Rodenhiser, et al., 2017; Fahrner & Bjornsson, 2019; Sobreira et al., 2017). Individuals with KS exhibit a highly sensitive and specific DNA methylation profile, or “episignature,” in blood that can be used as a biomarker to complement standard clinical diagnostic tools in cases with ambiguous clinical phenotypes or inconclusive genetic test results (Aref‐Eshghi et al., 2018; Aref‐Eshghi, Schenkel, Lin, Skinner, Ainsworth, Pare, Rodenhiser, et al., 2017). For example, episignatures have been used to reclassify variants of uncertain significance (VUSs) as likely benign or pathogenic in equivocal cases of KS (Aref‐Eshghi et al., 2018; Aref‐Eshghi, Bend, et al., 2019; Aref‐Eshghi, Bourque, et al., 2019; Aref‐Eshghi, Schenkel, Lin, Skinner, Ainsworth, Pare, Rodenhiser, et al., 2017).

Somatic mosaicism—the presence of two or more populations of cells with distinct genotypes in a single organism occurring post‐zygotically—has been detected in a subset of patients with classical clinical features of KS. A recent retrospective study of 12,000 individuals estimated that 1.5% of all molecular diagnoses were attributed to a mosaic variant detected in proband blood samples. Of those, mosaic variants in *KMT2D* represented 1.6% (2/120) of all detected mosaic variants in the analyzed cohort (Cao et al., 2019). To our knowledge, 11 individuals mosaic for *KMT2D* variants have been reported in the literature with alternate allele frequencies ranging from 10% to 37% in blood (Table 1) (Banka et al., 2013; Lepri et al., 2017; Manheimer et al., 2018; Murakami et al., 2020). Some showed milder phenotypes including average heights and potentially less distinctive facial features, and most (7/11) do not have congenital heart defects (Banka et al., 2013; Cao et al., 2019; Lepri et al., 2017; Manheimer et al., 2018; Murakami et al., 2020). However, 18% (2/11) had hypoplastic left heart with aortic and mitral atresia (HLHS MA‐AA; Table 1). Both individuals were mosaic for frameshift variants, in agreement with the 5%–13% of HLHS found among patients with KS (Digilio et al., 2017; Murakami et al., 2020).

**TABLE 1:** Clinical features of individuals with mosaic *KMT2D* variants

Clinical features	Proband 1	GM13‐3816 (Lepri et al., 2017)	KB450 (Lepri et al., 2017)	KB369 (Lepri et al., 2017)	1 (Banka et al., 2013)	2 (Banka et al., 2013)	3 (Banka et al., 2013)	22M (Cao et al., 2019)	23M (Cao et al., 2019)	KMS‐025 (Murakami et al., 2020)	1‐00596 (Manheimer et al., 2018)	1‐00479 (Manheimer et al., 2018)
*KMT2D* variant	c.8200C>T (p.R2734*)	c.15061C>T (p.R5021*)	c.13450C>T (p.R4484*)	c.3596_3597delTC (p.L1199Hfs*7)	c.9494delA (p.D3165Vfs*32)	c.8463_8475del (p.A2823Pfs*24)	Whole gene deletion	c.10938_10939delinsT (p.P3647Lfs*11)	c.8506C>T (p.R2836C)	c.15844C>T (p.R5282*)	c.5166del (p.S1722Rfs*9)	c.13727_13728del (p.F4576Cfs*29)
Alternate allele frequency in whole blood	11.2%	16%	34%	20%	<15%	Unable to quantify	10–20%	27.8%	10.4%	37%	33%	20%
Variant classification by ACMG guidelines	Path	Path	Path	Path	Path	Path	Path	Path	VUS	Path	Path	Path
Gender	M	F	F	F	F	F	F	NA	NA	M	NA	NA
Age in years (at publication)	1	6.1	8	17	11	6	10	NA	NA	NA	NA	NA
Elongated palpebral fissures	+	+	+	+	+	+	+	NA	NA	+	NA	NA
Sparse eyebrows	+	+	+	+	+	+	+	NA	NA	+	NA	NA
Palpebral ptosis	−	+	−	+	−	+	−	NA	NA	−	NA	NA
Broad nasal tip	+	−	+	+	+	+	−	NA	NA	+	NA	NA
Thin upper and full lower lip	+	+	+	+	+	+	+	NA	NA	+	NA	NA
Large dysmorphic ears	+	+	+	−	NA	NA	+	NA	NA	+	NA	NA
Fetal pads	+	−	+	+	+	+	+	NA	NA	+	NA	NA
Feeding difficulties	+	−	−	−	−	+	+	NA	NA	NA	NA	NA
Palate	High arched and V shaped	Normal	Normal	Normal	Normal	V‐shaped	Cleft lip and palate	Bilateral cleft lip and palate	NA	High arched palate	NA	NA
Cardiac defects	+	−	+	−	−	−	−	+	Not specified	−	+	+
HLHS	HLHS, aortic/mitral atresia, pulmonary vein stenosis	No	No	No	No	No	No	Not specified	Not specified	No	HLHS, Double aortic arch, aortic/mitral atresia	HLHS, aortic/mitral atresia
Urogenital anomalies	−	−	−	−	−	Right ectopic kidney	−	NA	NA	Cryptorchidism and multicystic dysplastic kidney	NA	NA
IQ impairment/development and learning	+	Borderline	Borderline	Moderate	Mild delay	Moderate delay	Severe, no speech	NA	Global developmental delay	Mild intellectual disability and developmental delay	NA	NA
Compromised adaptive functioning		Mild	On average	Mild				NA	NA	NA	NA	NA
Hypotonia in infancy	+		+	NA	−	+	+	NA	+	NA	NA	NA
Motor delay	+			NA	Mild	Yes	Mild	NA	NA	NA	NA	NA
Vision	Exopthalmos	−	−	−	Hypermetropia and right convergent squint	Hypermetropia and right convergent squint	Pale optic discs	NA	Congenital nystagmus	NA	NA	NA
Hearing	−	−	−	−	No problems	Decreased hearing at low frequencies. Sensitive to loud noises	No problems	NA	NA	NA	NA	NA
Joint dislocations	−	−	Right hip dysplasia	−	Congenital left hip dislocation needing open reduction	None	None	NA	NA	NA	NA	NA
Endocrine anomalies	−	−	−	Precocius puberty	Precocious puberty at 8	Postnatal hypoglycemia requiring dextrose infusion – resolved	None	NA	NA	NA	NA	NA
Head circumference	85th	10th	−	<50th	90th percentile	0.4th percentile	25–50th percentile	NA	Microcephaly	NA	NA	NA
Height	80th	75–90th	−	<25th	50–75th	50–75th	50–75th	NA	Short stature	NA	NA	NA
Weight	70–80th	97th	−	>50th	99th	91–98th	Not known	NA	NA	NA	NA	NA
Immunity related problems	Recurrent infections	−	−	−	Recurrent otitis media	Recurrent otitis media		NA	NA	Otitis media	NA	NA
Other	Inoperable heart disease	Pigmentary anomalies, body asymmetry	Diaphragmatic hernia	Generalized anxiety disorder, multiple phobias, autistic‐like behavior	Joint hyperextensibility with small lateral incisors	Constipation, broad thumbs and toes, anteriorly placed anus, irregularly spaced teeth	Hemangiomatous lesion on right thigh and epilepsy	Holoprosencephaly	Soft skin, abnormal facial shape	Lipoma	NA	NA

We present a case of an infant with HLHS MA‐AA, exophthalmos due to shallow orbits, and evolving facial features consistent with KS1 found to have a mosaic nonsense variant in *KMT2D* at a low allele fraction (11.2%). Given the somewhat nonclassical clinical presentation and low level of mosaicism in blood, we complemented trio whole exome sequencing with DNA methylation profiling, which together confirmed the diagnosis of KS1.

## 2 METHODS

### 2.1 DNA methylation data analysis

Methylation analysis was performed with the clinically validated EpiSign assay as previously described (Aref‐Eshghi et al., 2020; Aref‐Eshghi, Bend, et al., 2019; Aref‐Eshghi, Bourque, et al., 2019; Sadikovic et al., 2021). Briefly, methylated and unmethylated signal intensity generated from the EPIC array was imported into R 3.5.1 for normalization, background correction, and filtering. Beta values ranging from 0 (no methylation) to 1 (complete methylation) were calculated as a measure of methylation level and processed through the established support vector machine (SVM) classification algorithm for EpiSign disorders. The EpiSign Knowledge Database composed of over 5000 methylation profiles from reference disorder‐specific and unaffected control cohorts was utilized by the classifier to generate disorder‐specific methylation variant pathogenicity (MVP) scores. MVP scores are a measure of prediction confidence for each disorder, ranging from 0 (discordant) to 1 (highly concordant). For patients with full pathogenic mutations, a positive EpiSign classification typically involves MVP scores greater than 0.5 in combination with concordant hierarchical clustering and multidimensional scaling.

## 3 CASE REPORT

### 3.1 Clinical case

Our patient is a male who was born to nonconsanguineous parents from Honduras at 39 weeks' gestation via vaginal delivery. Fetal ultrasound at 27 weeks and 6 days revealed a hypoplastic nasal bone, and fetal echocardiogram showed a hypoplastic left heart with mitral and aortic atresia, which was confirmed on postnatal echocardiogram. Physical examination at birth was notable for normal muscle tone and respiratory effort with APGAR scores of 8 and 8. He was maintained on prostaglandin E infusion to maintain ductal patency and underwent stage 1 reconstruction with the Norwood Sano operation on his third day of life. At 3 months of age, he underwent cardiac catheterization with balloon angioplasty of a distal neo‐aortic coarctation. At 4 months, he had a stage 2 Glenn palliation and atrial septostomy with stem cell injection, after which he developed persistent chylous effusions. His left pulmonary artery was dilated at 7 months of age, and at 8 months, he underwent a sutureless pulmonary vein repair for stenosis of the pulmonary vein entry site into the left atrium. He developed respiratory insufficiency with left diaphragm paralysis that required plication. Persistent left‐sided and distal airway collapse required prolonged mechanical ventilation. Two months later, he developed severely elevated right ventricular filling pressures and mean Glenn circulation pressures, minimal to no forward blood flow into both upper lungs, and copious aortopulmonary collateral arterial flow that was treated with extensive coil embolization. He was not eligible for either stage 3 Fontan procedure or heart transplantation given his underlying cardiac complexity, degree of pulmonary vein stenosis, and chronic respiratory failure.

**FIGURE 1:**
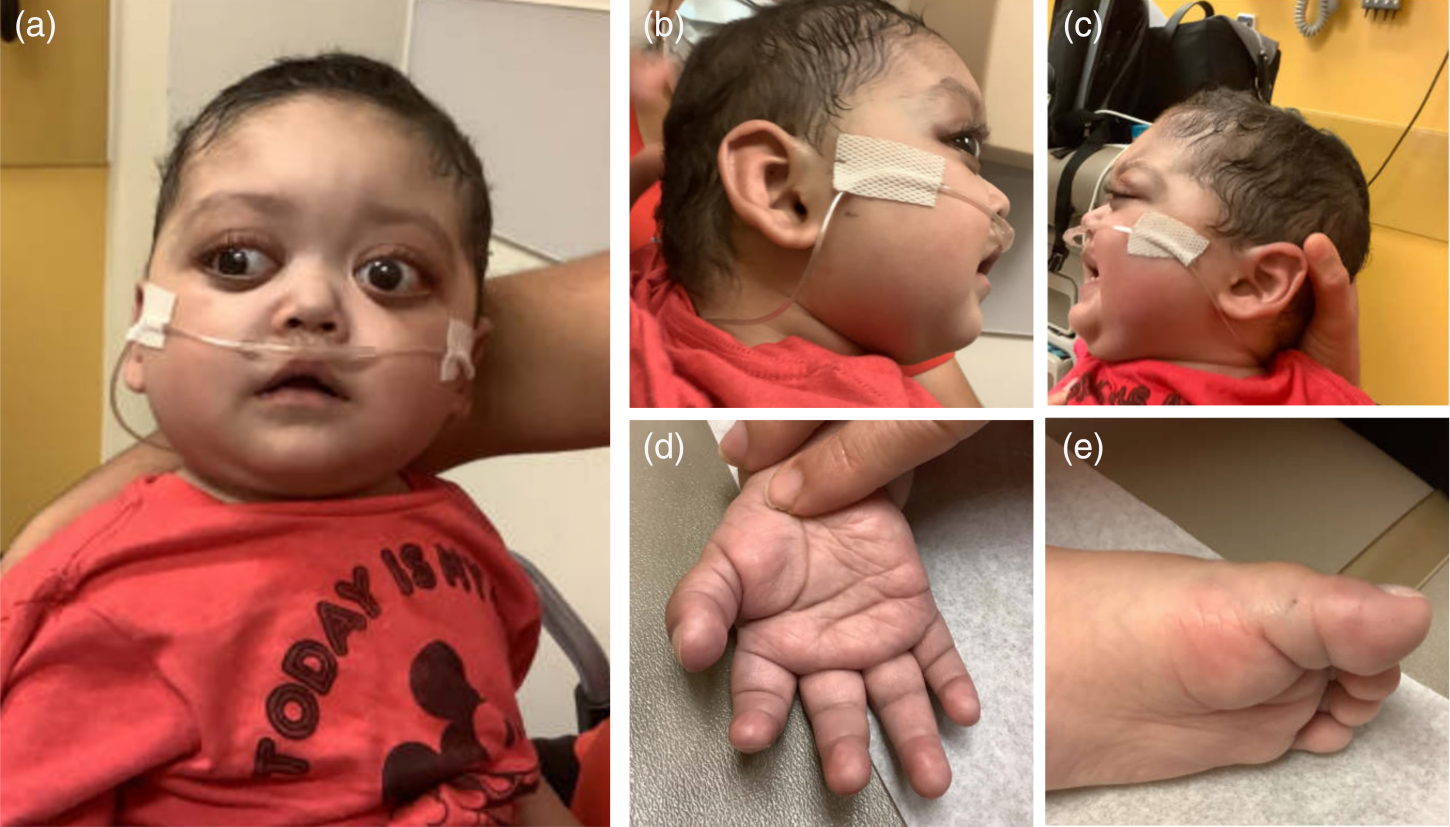
Clinical features of the patient described in this report at 13 months of age (4 months after initial evaluation). (a) Facial features include arched eyebrows with lateral sparseness, long palpebral fissures with eversion of lateral third of the lower eyelids, long eyelashes, broad nasal tip, short columella, and epicanthal folds. Unique features included exophthalmos due to shallow orbits, depressed nasal bridge, and a bulbous nose. (b, c) Lateral views illustrating prominent ears. Persistence of (d) fingertip and (e) toe pads

He had a distinct facial appearance, which was not fully appreciated initially due to his critically ill state. At 9 months of age, he had striking exophthalmos, prompting ophthalmology, endocrinology, and genetics evaluations. Exophthalmos was thought to be secondary to shallow orbits and not to Grave's disease. Thyroid function tests were consistent with sick euthyroid syndrome. Evaluation at 13 months of age in our Epigenetics and Chromatin Clinic revealed long palpebral fissures (>2 standard deviations [*SD*s], above the mean for age) with eversion of the lateral third of the lower eyelid, long eyelashes, and highly arched and broad eyebrows with notching and thinning of the lateral third that had not been noticed in the first inpatient genetics evaluation. He was brachycephalic and had prominent ears. He had a short nose and a short columella with a depressed nasal bridge and a broad nasal tip. He had a tented upper lip, a high V‐shaped palate, misaligned teeth, and persistent fingertip and toe pads (Figure 1).

The proband had significant neurological findings, which prompted an evaluation by pediatric neurology. He was diffusely hypotonic and had delayed developmental milestones, poor coordination, and persistent irritability. He had an abnormal EEG with diffuse slowing and possible mild asymmetry but no seizure activity. Head ultrasound and brain MRI showed no acute intracranial abnormalities with prominence of the bifrontal extra‐axial CSF spaces and lateral ventricles, scattered microhemorrhages commonly seen after cardiac surgery, and mild diffuse thinning of the corpus callosum.

His growth parameters (including weight, length, and head circumference) have been mostly within the typical range for age with a few exceptions when his weight and length temporarily dipped to more than two SDs below the mean for age. Contributing factors included oral motor dysfunction and feeding intolerance, which required nasogastric feedings and ultimately gastrostomy tube placement with Nissen fundoplication. His slow weight gain improved significantly after initiation of enteral feeding with fortified formula. His clinical course was also complicated by medical necrotizing enterocolitis (NEC) and a severe IV infiltrate in his left wrist.

Additional work up included an abdominal ultrasound which showed structurally normal abdominal organs with elevated resistive indices in the main renal arteries of the kidneys, likely secondary to known cardiac disease. Skeletal survey showed no abnormalities of the hips or vertebrae and was only notable for mild diffuse bony demineralization. He had a healthy 3‐year‐old sister, and there was no family history of congenital heart disease or other birth defects.

He was discharged home shortly after his first birthday, having spent his first 9 months at an outside hospital and the last 3 months at our institution. He went home on 0.5 L of oxygen by nasal canula, continuous G tube feeds, a regimen of diuretics that required enteral replacement of electrolytes, and aspirin for patency of his Glenn anastomosis. His outpatient management included close follow up with cardiology, pulmonology, and genetics, as well as a plan for sedation wean under hospice guidance. He was readmitted on several occasions to the cardiology service for increased work of breathing and increasing oxygen requirement in the setting of recurrent upper respiratory infections.

### 3.2 Genetic testing

FISH of uncultured amniocytes revealed XY, diploid chromosomes 13, 18, and 21. Reflex SNP microarray analysis was normal. A postnatal microarray did not detect copy number abnormalities. Clinical trio exome sequencing performed at GeneDx identified a de novo pathogenic variant in *KMT2D* (c.8200C>T, p.R2734X) present in 11.2% of the 223 sequencing reads from peripheral blood. He also had a maternally inherited likely pathogenic intragenic deletion involving at least exons 9–11 of the *BMPER* gene that is associated with autosomal recessive diaphanospondylodysostosis.

**FIGURE 2:**
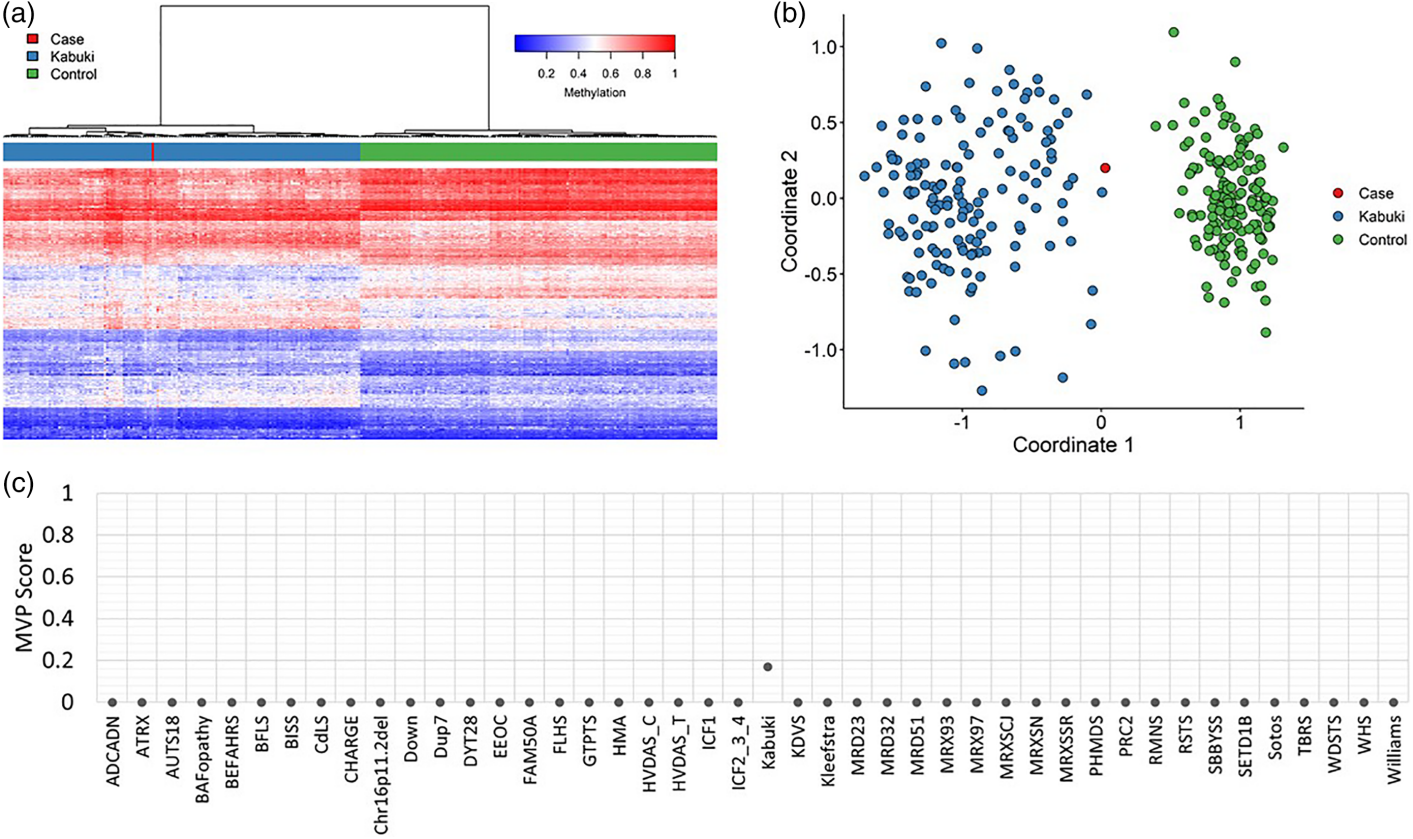
EpiSign DNA methylation analysis of peripheral blood from a patient with a somatic mosaic nonsense variant in *KMT2D*, the causative gene for Kabuki syndrome 1 (KS1). (a) Hierarchical clustering and (b) multidimensional scaling plots indicate that the patient (red) has a DNA methylation signature similar to subjects with a confirmed KS episignature (blue) and distinct from controls (green). Each row of the heatmap represents one CpG probe on the DNA methylation array, and each column represents one individual's sample. The heatmap color scale from blue to red represents the DNA methylation level (beta value) from 0 (no methylation) to 1 (fully methylated). (c) MVP score, a multiclass supervised classification system capable of discerning between multiple episignatures by generating a probability score for each episignature. The elevated patient score for KS compared to other syndromes suggests an episignature similar to the KS reference signature; however, the decreased probability compared to what is typically observed for constitutional KS (score >0.5) likely results from the low‐level mosaicism of the variant

EpiSign variant‐targeted analysis performed at Greenwood Genetic Center revealed a genome‐wide DNA methylation profile consistent with KS (Figure 2a–c). EpiSign analysis was concordant with a methylation signature observed in patients with *KMT2D* mutations, including Euclidean clustering (Figure 2a) and multidimensional scaling (Figure 2b); however, MVP score (Figure 2c) showed only a moderate MVP elevation (0.17) which may be reflective of the mosaic status of the mutation in this patient.

## 4 DISCUSSION

The patient reported in this study presented with HLHS MA‐AA, pulmonary vein stenosis, hypotonia, developmental delay, and feeding difficulties. His craniofacial features included long palpebral fissures (>2 *SD* above the mean for age) with eversion of the lateral third of the lower eyelid, arched, and broad eyebrows with notching and lateral sparseness, a short columella, prominent ears, and persistent fingertip and toe pads, meeting definite diagnostic clinical criteria for KS (Adam et al., 2019). His developmental delay could be attributable to being hospitalized and critically ill for most of his life. In addition, he also had exophthalmos due to shallow orbits and a short nose with depressed nasal bridge less typical of KS. Whole exome sequencing revealed low‐level mosaicism for a de novo pathogenic variant in *KMT2D* (allele frequency of 11.2%). Congenital heart disease has been detected in 70% of patients with *KMT2D* pathogenic variants, including 47% with left sided obstructive lesions (mostly aortic coarctation and bicuspid aortic valve). Among patients with KS, HLHS has been reported in 5%–13% of cases (Digilio et al., 2017; Murakami et al., 2020). The severity of his heart disease and low allele frequency found in peripheral blood prompted us to confirm the diagnosis of KS1 using the EpiSign genome‐wide DNA methylation assay. Such episignatures have been used to diagnose KS in individuals with suggestive phenotype but negative or inconclusive genetic testing and to reclassify variants of uncertain significance as likely pathogenic or benign (Aref‐Eshghi et al., 2018; Aref‐Eshghi, Bend, et al., 2019; Aref‐Eshghi, Bourque, et al., 2019; Aref‐Eshghi, Schenkel, Lin, Skinner, Ainsworth, Pare, Rodenhiser, et al., 2017). At this time, episignatures have only been established in blood.

Mosaic variation poses a significant challenge for disease prognosis and accurate prediction of future manifestations. Whereas mosaic variation is known to cause disease, recognizing underlying mosaicism is challenging without overt cutaneous manifestation or when phenotypes deviate from what has been reported in patients with the nonmosaic variant (Biesecker & Spinner, 2013). Fortunately, the widespread use of exome sequencing has facilitated the detection of low‐level mosaicism in blood (Gajecka, 2016). Approximately 1.5% of all molecular diagnoses from a cohort of 12,000 individuals were attributed to a mosaic variant detected in the proband (Cao et al., 2019). Individuals with KS1 resulting from pathogenic mosaic frameshift *KMT2D* variants described thus far have presented with typical to more mild facial features and have varying degrees of cardiovascular involvement. Of those, HLHS has been described in 18% (2 out of 11 cases; see Table 1) (Banka et al., 2013; Cao et al., 2019; Lepri et al., 2017; Manheimer et al., 2018; Murakami et al., 2020).

The proportion of variants shared across tissues is predicted to vary according to the stage of embryonic development at which the variant arose. It is often difficult to pinpoint precisely when a particular pathogenic variant occurred during development, and different mosaic ratios across tissues can affect the phenotypic presentation. Variants acquired later in development are predicted to have lower alternative allele frequencies in peripheral blood (Ju et al., 2017). We detected a somatic variant affecting heart development using peripheral blood, a tissue that is also derived from mesoderm. We therefore suspect that constitutional somatic mosaicism is responsible for the congenital heart defect and other systemic manifestations of KS1 in the proband, although we did not have tissue available to confirm the level of cardiac mosaicism.

We used DNA methylation profiling as an orthogonal method to confirm the diagnosis and observed a highly specific KS1 episignature, thus expanding the clinical utility of this method. EpiSign is validated to diagnose over 40 neurodevelopmental genetic conditions (including KS) and as a functional assay to resolve variants of uncertain significance in genes with a known epigenetic signature (Aref‐Eshghi et al., 2020; Aref‐Eshghi, Bend, et al., 2019; Aref‐Eshghi, Bourque, et al., 2019; Sadikovic et al., 2019). Episignatures have been used to detect mosaicism in imprinting disorders (Aref‐Eshghi, Schenkel, Lin, Skinner, Ainsworth, Pare, Siu, et al., 2017) and Fragile X syndrome at levels greater than 20% (Schenkel et al., 2016). Interestingly, Aref‐Eshghi et al. described a case of an affected individual with a rare intronic variant in *KMT2D* and the previously described KS episignature whose mother had a variant allele fraction of 7% and a normal DNA methylation profile (Aref‐Eshghi, Bend, et al., 2019; Aref‐Eshghi, Bourque, et al., 2019). This observation, along with our findings reported here of an individual with a variant allele fraction of 11.2% exhibiting a KS episignature, suggests that DNA methylation profiling can detect the KS epigenetic signature in blood when mosaicism is present at levels of 7%–11%. Therefore, in cases of suspected KS—and other disorders with established episignatures—if low‐level mosaicism is identified through sequencing, genome‐wide DNA methylation profiling should serve as the next step in diagnosis. We favor the use of this complementary genome–epigenome approach for other conditions with ambiguous phenotypes and elusive molecular diagnoses, such as distinct Mendelian disorders of the epigenetic machinery and imprinting disorders.

Our report adds to the published spectrum of *KMT2D* mosaic phenotypes and suggests that in some cases making a diagnosis of KS warrants high‐depth sequencing coverage of *KMT2D* to uncover potential mosaicism. Ours is the first report demonstrating the utility of DNA methylation profiling as a valuable functional diagnostic test in cases of KS with ambiguous phenotypes and low‐level mosaicism. This case provides yet another example illustrating the power of utilizing combinatorial genomics and epigenomics approaches to unambiguously identify and interpret different types of genetic variation in order to optimize diagnostic capabilities.

## CONFLICT OF INTEREST

The authors declare that there is no conflict of interest that could be perceived as prejudicing the impartiality of the research reported.

## AUTHOR CONTRIBUTIONS

Carolina Montano collected data and wrote the article. Jill A. Fahrner and Nara Sobreira led the study and revised the article. Jill A. Fahrner facilitated the external collaboration and oversaw data collection. Nara Sobreira, Jacqueline R. Harris, and Jacquelyn F. Britton contributed to conceptualization, clinical care, consenting, and supervision. Benjamin T. Barnes contributed to clinical care and evaluation of cardiac phenotype. Jennifer Kerkhof and Bekim Sadikovic analyzed data and critically evaluated the article. Jennifer A. Lee generated data. All authors reviewed and approved the article.

## Data Availability

The data that support the findings of this study were obtained from clinical laboratories. Restrictions apply to the availability of these data, which are not publicly available due to privacy and ethical restrictions.
